# Concurrent Presentation of Hodgkin Lymphoma and Multiple Myeloma

**DOI:** 10.1155/2013/398769

**Published:** 2013-08-20

**Authors:** Rekha Chandran, Miklos Simon, Stephen E. Spurgeon

**Affiliations:** ^1^Department of Hematology and Oncology, Medical College Wisconsin, 8701 Watertown Plank Road, Milwaukee, WI 53226, USA; ^2^Compass Oncology, 5050 NE Hoyt Street, Suite 256, Portland, OR 97213, USA; ^3^Knight Cancer Institute, Oregon Health Sciences and University, 3181 SW Sam Jackson Park Road, Portland, OR, USA

## Abstract

The simultaneous presentation of the Hodgkin lymphoma and multiple myeloma in the absence of prior chemotherapy or radiation is very rare. Here, we discuss a 72-year-old patient who initially presented with generalized pruritis. Workup led to a diagnosis of multiple myeloma which progressed and required treatment. As part of his pretreatment workup, an MRI was performed to evaluate skeletal lesions. This revealed diffuse and bulky adenopathy which was confirmed by PET. A biopsy of an axillary node was consistent with the nodular sclerosing type Hodgkin lymphoma. He was treated with adriamycin, bleomycin, vinblastine, and dacarbazine (ABVD) chemotherapy × 6 resulting in complete resolution of his adenopathy and pruritis as well as improvement in his myeloma.

## 1. Introduction

The simultaneous presentation of the Hodgkin lymphoma (HL) and multiple myeloma (MM) in the absence of prior chemotherapy is very rare. We discuss a patient with concurrent presentation of both malignancies.

## 2. Case Presentation

A 72-year-old male with a past history of hypertension and depression presented to his primary care provider with generalized pruritus for six months. He did not report fever, night sweats, or weight loss. There was no skin rash, lymphadenopathy, or hepatosplenomegaly on exam. Blood tests showed no evidence of anemia, renal failure, or hypercalcemia. A trial of treatment with antihistamines and topical steroids failed to provide relief, and his clinical symptoms as well as exam remained unchanged. A more extensive panel of tests was ordered to look for systemic illness including underlying malignancies. Among these, serum protein electrophoresis with immunofixation was notably abnormal and showed a kappa restricted IgG monoclonal protein on (3 g/dL). 24-hour urine collection showed the presence of the Bence Jones protein (5.9 mg/dL) and trace of monoclonal free light chain protein. Given the concern for MM, he was referred to a hematologist for further workup. Bone marrow showed 8% plasma cells but no abnormalities on skeletal survey. Beta-2 microglobulin (B2M) was elevated at 3 mg/L (1.1–2.4 mg/L). Based on these initial results, he was diagnosed with smoldering multiple myeloma and placed on active surveillance.

After four months of followup, the patient continued to complain of pruritus unresponsive to symptomatic treatment. Repeat lab testing done at that time revealed increased monoclonal IgG (3.65 g/dL) and anemia (hemoglobin of 12 gm/dL). A repeat bone marrow biopsy showed significant increase in monoclonal plasma cells from 8% to 30%. Cytogenetic and fluorescent in situ hybridization (FISH) studies were normal. Renal function and calcium levels were normal. A bone survey revealed osteoporosis. A diagnosis of multiple myeloma stage II (International Staging System) was made, and he was referred to a tertiary care cancer center for treatment recommendations. Treatment with bortezomib and dexamethasone followed by autologous transplantation was recommended. As part of his pretreatment workup, a magnetic resonance imaging (MRI) of the spine was performed. No skeletal lesions were identified; however, the MRI revealed abdominal adenopathy. A subsequent positron emission tomography (PET) showed bulky PET avid adenopathy with a nodal mass extending from the left supraclavicular region to the left axillary region measuring 15 × 7 × 4 cm. Enlarged portocaval nodes measuring 4.2 × 2.3 cm and retroperitoneal left paraaortic lymph node measuring 3.3 × 2.2 cm were also noted.

Axillary lymph node biopsy was performed and showed nodular sclerosing type Hodgkin lymphoma (HL), and treatment with multiagent chemotherapy was recommended. Of note, during his ongoing evaluation and prior to completion of his workup, the patient did receive treatment with one cycle of bortozemib plus dexamethasone. However, after the diagnosis of HL was made, this was stopped and he was treated with adriamycin, bleomycin, vinblastine, and dacarbazine (ABVD) chemotherapy × 6 cycles administered every 4 weeks. 

He tolerated treatment well, and after two cycles his pruritus completely resolved. An interim and posttreatment follow-up PET showed no residual hypermetabolic activity consistent with complete remission. Bone marrow biopsy at the completion of treatment for HL also showed a significant reduction of his monoclonal plasma cells and a significant reduction in his IgG and normalization of  his B2M (Table 1). His laboratory tests are summarized in the Table 1.

Given the significant improvement in his MM, no additional MM therapy was pursued, and the patient continued active surveillance. Three years after ABVD, he did not have evidence of MM progression or recurrence of HL.

## 3. Discussion

Concurrent presentation of MM and HL in the absence of prior radiation or chemotherapy is rare and limited to case reports [1–5]. These patients are usually older than 50 which is typically the second age peak for HL incidence. MM is also usually a disease of older individuals. The cell of origin for both HL and MM is the B lymphocyte and the simultaneous presentation may reflect multilineage transformation of neoplastic B cells [1].

Case reports have linked generalized pruritus to almost every type of malignancy. In the absence of clinically evident lymphadenopathy as in our patient's case, physicians are more likely to order diagnostic workup directed towards MM due to patient's advanced age. However, pruritus as a manifestation of MM is exceedingly rare. It has been described in a few cases and when myeloma is associated with liver disease, amyloidosis, and renal disease [6, 7]. On the other hand, persistent generalized pruritus may be seen in up to 30% of patients with HL, particularly the nodular sclerosing type with mediastinal mass [8]. It may predate the diagnosis by up to 5 years and was considered a B symptom from 1965 to 1971. Fever replaced pruritus as a B symptom as the latter did not have a predictable impact on survival [8]. From a diagnostic point, increased plasma cells in the bone marrow may be seen with HL irrespective of the stage of the disease and is considered reactive, but significant bone marrow plasmacytosis is suspicious for concurrent MM [1]. Although the initial outpatient workup for pruritus in our patient led to the diagnosis of MM, the chief complaint resolved only after diagnosis and treatment of lymphoma. Despite its rarity, it is important for physicians to consider the possibility of concurrent HL and MM particularly in older patients who present with symptoms such as pruritus that are atypical for myeloma.

## Key Points

 (i) Concurrent presentation of the Hodgkin lymphoma and multiple myeloma is extremely rare and limited to case reports.
 (ii) Generalized pruritus may be seen in up to 30% of individuals with HL with mediastinal involvement but is extremely uncommon in MM.
 (iii) It is important for physicians to consider the possibility of a second malignancy especially in older individuals who present with symptoms that are not typical of the initially diagnosed malignancy.

## Figures and Tables

**Table 1:** Summary of serial lab results.

Tests	Initial	Four months later	Completion of treatment
IgG level (gm/dL)	3	3.65	1.1
Beta-2 microglobulin (mg/L)	3.0	3.5	1.5
Bone marrow biopsy	8% plasma cells	30% plasma cells	15%
24 hr urine protein electrophoresis	Trace monoclonal free light chain	21 mg	None
